# Pharmacist and Physician Collaborative Practice Model Improves Vancomycin Dosing in an Intensive Care Unit

**DOI:** 10.4236/ijcm.2016.710073

**Published:** 2016-10-25

**Authors:** Dimitriy Levin, Jeffrey J. Glasheen, Tyree H. Kiser

**Affiliations:** 1 Department of Medicine, University of Colorado, Aurora, Colorado, USA; 2 University of Colorado Hospital Medicine Group, Aurora, Colorado, USA; 3 University of Colorado Hospital Authority, Aurora, Colorado, USA; 4 Department of Clinical Pharmacy, University of Colorado Skaggs School of Pharmacy and Pharmaceutical Sciences, Aurora, Colorado, USA

**Keywords:** Vancomycin, Nomogram, Protocol, Collaborative Practice, Therapeutic Drug Monitoring

## Abstract

**Objective:**

A pharmacist and physician collaborative practice intervention to improve the initial dosing of vancomycin was implemented with the goal of decreasing the number of subtherapeutic first troughs and increasing the number of therapeutic troughs.

**Methods:**

Using the best available evidence, a nomogram was created to determine the initial vancomycin dose. The nomogram utilized actual bodyweight and glomerular filtration rate (eGFR) estimated with the MDRD4 equation. The dose was based on the 2009 ASHP/IDSA/SIDP guidelines, which recommended 15–20 mg/kg every 8–12 hours. Providers ordered “vancomycin IV dosed per pharmacy”.

**Results:**

The pre- (n = 75) and post-intervention (n = 108) cohorts had similar age, gender distribution, weight, and eGFR. The median total daily vancomycin dose was similar in pre- and post-intervention groups (2000 mg), although the median first trough was higher following the intervention (13.0 vs. 14.8 mcg/ml, p = 0.03). Following the intervention, the proportion of first troughs under 10 mcg/ml decreased (32% to 13%, *p* = 0.003), while the proportion of troughs in the 10 – 20 mcg/ml therapeutic range increased (50.7% vs. 69.4%, *p*= 0.01). There was no difference in the proportion of troughs over 20 mcg/ml (17.3% vs. 17.6%, *p*= 0.96).

**Conclusions:**

A multi-disciplinary intervention utilizing a nomogram-based pharmacy collaborative practice model significantly improves the proportion of therapeutic initial vancomycin troughs and decreases the number of subtherapeutic troughs by half.

## 1. Introduction

Vancomycin is a glycopeptide antibiotic with activity against a variety of Gram-positive organisms, including methicillin-resistant *Staphylococcus aureus* (MRSA). It is commonly used as part of an empiric broad-spectrum antimicrobial regimen in critically ill patients. Vancomycin must be given intravenously when used for systemic infections, with dose adjustment for body weight and renal function [1].

Although there is a dearth of high-quality data on optimal dosing strategies for vancomycin, in 2009 the American Society of Health-System Pharmacists (ASHP), the Infectious Diseases Society of America (IDSA), and the Society of Infectious Diseases Pharmacists (SIDP) released joint consensus recommendations based on the best available evidence [1]. The expert panel recommended monitoring of steady-state vancomycin troughs with a goal level above 10 mcg/ml to avoid development of resistance. No preference was given to intermittent versus continuous dosing.

Vancomycin typically takes 36 – 48 hours to reach a steady state. If the initial dose is incorrect, a patient may be severely under- or overdosed for a considerable period of time with risk of treatment failure, development of antimicrobial resistance, or vancomycin toxicity. To mitigate this, several nomograms have been published, targeting troughs greater than 5 mcg/ml [2] and 7.5 mcg/ml [3]. However, these nomograms were published before the emergence of heteroresistant and vancomycin-intermediate *Staphylococcus aureus* (VISA) strains and the publication of joint consensus recommendations to keep troughs over 10 mcg/ml. The upper trough limit recommended in literature is 20 mcg/ml based on the American Thoracic Society (ATS) and IDSA guidelines on hospital-acquired pneumonia [4] and IDSA guidelines on bacterial meningitis [5].

Given the interpatient variability and complexity of vancomycin dosing in intensive care unit patients, utilizing a multidisciplinary approach to therapy could improve time to therapeutic target attainment and patient safety. Pharmacists have specialty training in pharmacokinetics and pharmacodynamics and have demonstrated improvements in drug utilization and patient outcomes in outpatient collaborative practice models [6] [7]. However, most inpatient studies evaluate the impact of pharmacists rounding with a multidisciplinary team [8], with few published studies evaluating inpatient pharmacy collaborative practice endeavors that fully delegate drug therapy management to the pharmacist.

We undertook a quality improvement initiative at our institution to optimize initial vancomycin dosing using a nomogram and a pharmacy collaborative practice approach. The primary objective was to decrease the number of subtherapeutic troughs due to antibiotic under dosing.

## 2 Methods

The study was performed at the University of Colorado Hospital, a tertiary academic hospital. The study protocol was approved by the Colorado Multiple Institutional Review Board. Patient consent was not required and a Health Insurance Portability and Accountability Act waiver was obtained.

### 2.1. Vancomycin Nomogram Design

The nomogram was designed using current recommendations to dose vancomycin at 15 – 20 mg/kg every 8–12 hours in patients with normal renal function (Table 1) [1]. The decision to use intermittent rather than continuous dosing was based on the lack of definitive benefit with the latter, balanced by increased complexity and logistics such as the need for dedicated intravenous access in patients already receiving continuous infusions of vasopressors, sedatives, and analgesics. Actual body weight was used for dosing, including in obese patients, based on the best current evidence [9]. Although doses up to 6000 mg/day have been reported in literature [10], we capped the maximum initial dose at 4500 mg/day (1500 mg every 8 hours) for safety. Since vancomycin clearance is strongly tied to renal function [3], dosing every 8 hours was prescribed for patients with very high estimated glomerular filtration rate (eGFR). The eGFR was calculated using the 4-variable Modification of Diet in Renal Disease (MDRD4) [11] equation using gender, age, creatinine, and race [eGFR = 186 × [Serum Creatinine (mg/dL)]^−1.154^ × Age^−0.203^ × (0.742 if Female) × (1.210 if African American)]. Vancomycin doses were rounded to 250 mg increments for dosing convenience. Therapeutic trough range was defined as 10 – 20 mcg/ml. This trough range was designed to yield an Area Under the Curve to Minimum Inhibitory Concentration (AUC:MIC) ratio of > 400 in the majority of patients, because the *Staphylococcus aureus* MIC at the University of Colorado Hospital is typically ≤1 mcg/ml.

### 2.2. Collaborative Practice Implementation

The vancomycin nomogram and the collaborative practice protocol were reviewed and approved by the University of Colorado Hospital Pharmacy and Therapeutics committee and were implemented per the Colorado State Boards of Medicine and Pharmacy collaborative practice model agreement. All clinical pharmacists involved in care of ICU patients were trained in nomogram use with a mandatory online educational module. Prescribing providers in ICUs were given a copy of the protocol and instructed to order “vancomycin IV dosed per pharmacy” rather than indicate dose and frequency, although providers were also allowed to override the protocol and order a different dose at their discretion. A clinical pharmacist then gathered the required demographic and laboratory information, calculated eGFR, and ordered the vancomycin dose from the nomogram. Additionally, the pharmacist ordered a vancomycin trough at an appropriate time prescribed per the protocol. Vancomycin pharmacokinetic tracking forms were reviewed on a weekly basis during the study period.

### 2.3. Patient Population

Adult patients started on vancomycin in the intensive care unit (ICU) were included regardless of body weight and renal function, including renal replacement therapy. Patient demographics, actual bodyweight, vancomycin dose, and vancomycin trough data was obtained from pharmacokinetic tracking forms filled out prospectively by ICU clinical pharmacists. Only patients without a measured vancomycin trough concentration were excluded from the study. Patients were divided into historical control and intervention groups. Historical data was obtained from records of patients treated during the 3 months prior to protocol initiation. Patients in the intervention group were treated under the collaborative practice protocol for the 6-month period post protocol initiation.

### 2.4. Outcomes Evaluated

The primary outcome of this study was the incidence of subtherapeutic initial vancomycin trough concentrations, defined as a first drawn steady-state trough value <10 mcg/ml [1]. Secondary outcomes evaluated were the percentage of initial trough concentrations in goal range (10 – 20 mcg/ml), percentage of supratherapeutic trough concentrations (>20 mcg/ml), and the median daily vancomycin dose. Outcomes were compared between historical controls and the intervention group.

### 2.5. Statistical Analysis

Assuming subtherapeutic values in 34% of vancomycin trough concentrations (< 10 mcg/ml) in the historical control group, a total of 70 patients were needed in each group to have an 80% power to detect a 20% absolute decrease in subtherapeutic vancomycin trough concentrations.

Statistical analysis of the results was performed with Microsoft Excel (Microsoft, Redmond, WA) and Statext 1.4.1 (Statext.com). Continuous variables were compared using the Mann-Whitney U test and the Kruskal-Wallis one-way analysis of variance. Categorical variables were compared using the Fisher’s exact test and the chi-square test for independence. All tests were 2-tailed. A p value less than 0.05 was considered statistically significant.

## 3. Results

### 3.1. Patient Characteristics

A total of 183 patients were enrolled in this study: 108 in the collaborative practice group and 75 in the historical control group. The suggested nomogram dosing regimen was used in 108/178 (60.1%) of eligible patients during the intervention period. Demographics of the 75 historical controls and the 108 patients dosed under the collaborative practice agreement are shown in Table 2.

### 3.2 Evaluation of Vancomycin Trough Concentrations

**Figure 1.**
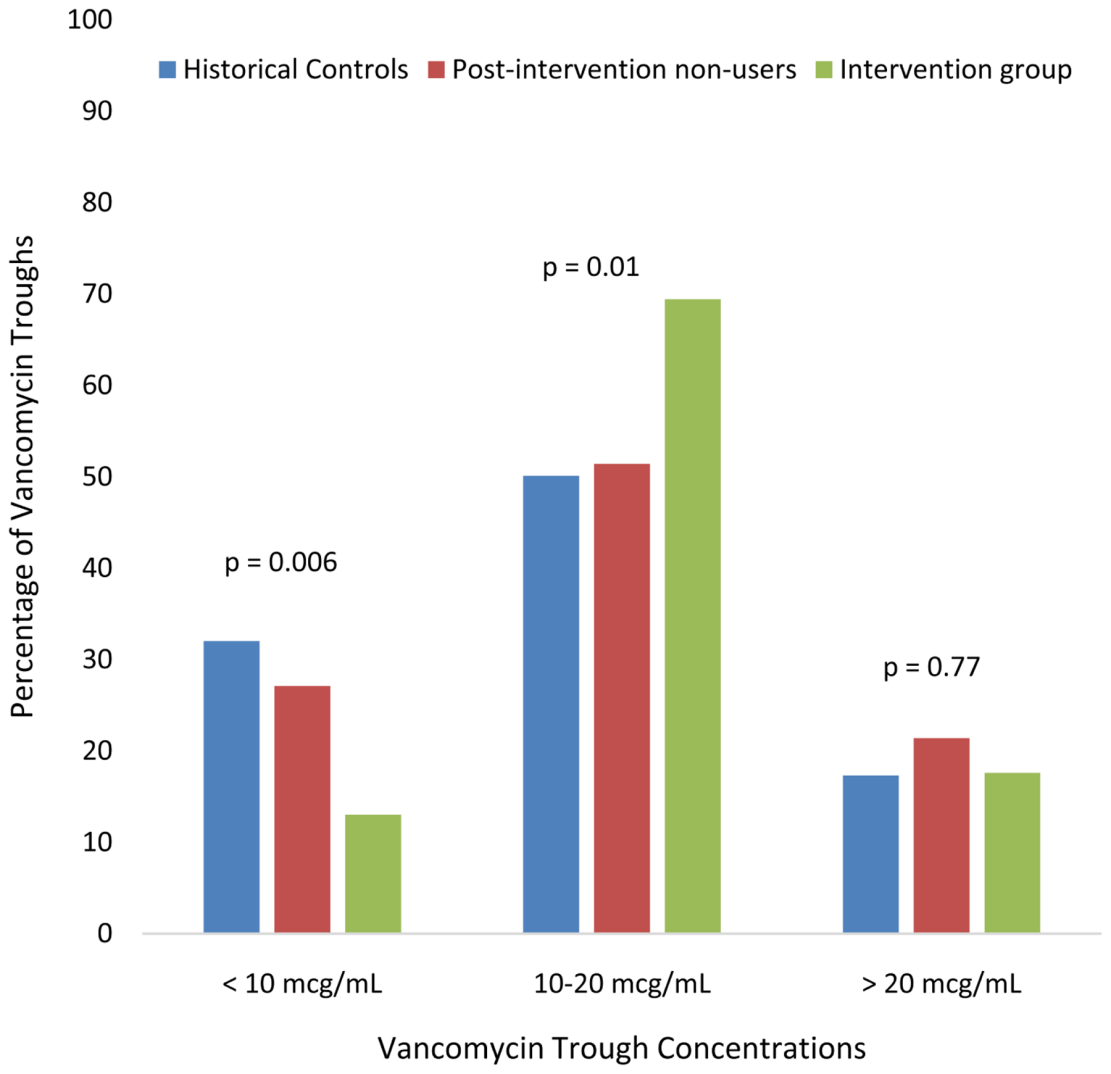
Distribution of initial vancomycin troughs among historical controls, patients not dosed per nomogram during the intervention period (non-users), and patients dosed using the collaborative intervention nomogram.

Although there was no statistically significant difference in the median total daily dose of vancomycin, the intervention group had a significantly higher median initial trough (14.8 mcg/ml, IQR 11.6 – 18.2, vs. 13.0 mcg/ml, IQR 9.1 – 16.8, *p*= 0.03). The intervention group experienced a significant reduction in the proportion of subtherapeutic troughs <10 mcg/ml (32.0% to 13.0%, *p* = 0.003), a significant increase in the number of in-range troughs 10 – 20 mcg/ml (50.7% to 69.4%, *p* = 0.01), and no significant change in supratherapeutic troughs > 20 mcg/ml (17.3% to 17.6%, *p* = 0.96) compared to historical control patients (Figure 1). The 70 patients who were not dosed using the collaborative practice option during the intervention period had a trough distribution similar to historical controls (27.1% low, 51.4% in-range, and 21.4% high; *p* = 0.02 for proportion of low and in-range groups compared to the collaborative practice nomogram cohort, *p* = 0.5 for comparison of supratherapeutic groups).

### 3.3. Evaluation of Vancomycin Troughs in Weight and eGFR Subgroups

The nomogram also performed well in subsets of patients at the extremes of weight and renal function (Table 3). Compared to historical controls and collaborative practice non-users, the intervention group had more patients with therapeutic troughs among those weighing 60 – 80 kg *(p* = 0.01). There was a trend towards significant improvement in therapeutic troughs in subgroups with body weight under 60 kg *(p* = 0.05) and eGFR > 90 ml/min/1.73 m^2^ (*p*= 0.08).

## 4. Discussion

We have demonstrated that a physician and pharmacist collaborative practice-based quality improvement initiative utilizing an evidence-based nomogram can successfully improve initial vancomycin dosing in critically ill patients. Our primary objective was to minimize the risk of underdosing and subsequent promotion of antimicrobial resistance, and in that respect 87% of patients dosed with the nomogram had an initial trough ≥10 mcg/ml. Although our nomogram was only 69% successful in reaching the therapeutic range of 10 – 20 mcg/ml, this is significantly better than provider-initiated dosing at our institution (44% - 51%) and in published studies of non-nomogram dosing (34%) [12]. While another previously published nomogram was 94% accurate [2], it targeted a much broader trough range of 5 – 20 mcg/ml. Accuracy of our protocol might have been adversely affected by the dynamic nature of ICU patients. Vancomycin clearance is strongly tied to renal function and since critically ill patients often have dynamic renal function, pinpointing an accurate eGFR can be challenging.

We observed a significant increase in the median vancomycin troughs without a corresponding change in the median total daily dose, as well as a decrease in subtherapeutic troughs without a concurrent increase in supratherapeutic troughs. This supports that patients were dosed more correctly for their bodyweight and renal function rather than simply receiving a higher dose across the board. The fact that provider-initiated dosing during the intervention period did not significantly differ from the historical controls favors the improvement being due to implementation of the nomogram.

It is important to note that our historical and non-user groups significantly differed from the nomogram cohort in certain weight subgroups. It is unclear whether this reflects our small sample size, a seasonal variation in ICU patients, or both. Unfortunately, analysis of all subgroups was limited by small sample size and a Type II error cannot be ruled out. Nevertheless, our data suggest improvement due to use of the nomogram in patients weighing less than 80 kg, and a trend toward significance with eGFR > 90 ml/min/m^2^.

Additional limitations of this study include a small sample size, application at a single hospital, and lack of clinical and microbiologic outcome data. *Staphylococcus aureus* MICs at our institution are typically ≤1 mcg/ml, so our nomogram was not directly designed to achieve the narrower trough serum concentration of 15 – 20 mcg/ml in all patients. Patients were only enrolled from the medical intensive care unit, so the applicability of our nomogram to other patient populations is unknown.

A large number of patients during the intervention period were dosed without the nomogram. This reflects the inherent challenge of implementing quality improvement initiatives that rely on changing behavior. Prior to the collaborative practice model, we attempted to educate providers to directly use the nomogram with a nearly zero-use rate despite frequent personal communication with physicians. Following roll-out of the collaborative practice, providers chose to bypass this process even though ordering “vancomycin per pharmacy” was easier than ordering a specific dose. We do not know if this was done for reasons of clinical judgment or because they did not know that this option was available. If clinical judgment was the reason for non-adherence, our findings of the non-nomogram group performing comparably to controls identify a potential knowledge and skill gap. Although clinical pharmacists were encouraged to call providers to encourage them to use the nomogram, this did not reliably happen because of workload issues and logistics.

In the course of this project, we were reminded of the scarcity of high-quality data on vancomycin dosing. With the advent of vancomycin-intermediate heteroresistant pathogens, a minimum trough of 15 mcg/ml may be required to avoid treatment failures. The narrow therapeutic range of 15 – 20 mcg/ml would be exceedingly difficult to attain even with the best dosing practices and high-quality research is urgently needed to establish the safe upper limit for vancomycin doses using the modern formulation of the antibiotic.

Finally, this study demonstrates that a physician and pharmacist collaborative practice model lends itself well to medications with complex dosing and monitoring requirements and should be explored as an effective quality improvement solution.

## 5. Conclusion

Utilization of an inpatient pharmacy collaborative practice model to manage vancomycin therapy resulted in a significant reduction in subtherapeutic trough concentrations and increased the percentage of therapeutic trough concentrations. This was accomplished without increasing the percentage of supratherapeutic trough concentrations. This inpatient multidisciplinary collaborative practice model should be evaluated in broader drug categories and patient populations to ensure reproducibility of these findings.

## Figures and Tables

**Table 1.** Vancomycin nomogram.

eGFR (ml/min/1.73 m)	Actual bodyweight (kg)			
	<60	60 – 80	81 – 100	>100
>90	750 mg q8	1000 mg q8	1250 mg q8	1500 mg q8
50 – 90	750 mg q12	1000 mg q12	1250 mg q12	1500 mg q12
15 – 49	750 mg q24	1000 mg q24	1250 mg q24	1500 mg q24
<15 or RRT	750 mg ×1	1000 mg ×1	1250 mg ×1	1500 mg ×1
**Therapeutic drug monitoring**				
For patients dosed every 8 – 12 hours, check trough 30 minutes prior to 4th dose For patients dosed every 24 hours, check trough 30 minutes prior to 3rd dose				
**Patients with eGFR < 15, continuous RRT, or unstable renal function**				
Give a one-time dose per nomogram Check a random vancomycin level 24 hours after the dose If random level is <20 mcg/mL, repeat dose If random level is >20 mcg/mL, do not redose, repeat random level in 12 hours				
**Patients on intermittent hemodialysis**				
Give a one-time dose per nomogram Check a random vancomycin level 2 hours after hemodialysis If random level is <20 mcg/mL, repeat dose If random level is >20 mcg/mL, do not redose, repeat level after next dialysis				

Dosing frequency is in hours (q8 = every 8 hours; ql2 = every 12 hours; q24 = every 24 hours). eGFR = estimated glomerular filtration rate; RRT = renal replacement therapy.

**Table 2.** Patient characteristics at vancomycin initiation.

Variable	Controls	Non-users	Intervention	*p*-value
	(n = 75)	(n = 70)	(n = 108)	
Age (years)	56 (47 – 64)	52 (39 – 62)	53 (42 – 64)	*p* = 0.4
Male sex (%)	56	59	56	*p* = 0.9
Actual bodyweight (kg)	85 (66 – 97)	88 (64 – 107)	78 (67 – 96)	*p* = 0.7
<60 kg	13%	20%	10%	*p* = 0.2
60 – 80 kg	25%	20%	46%	*p* < 0.005
81 – 100 kg	40%	29%	21%	*p* = 0.02
>100 kg	21%	31%	22%	*p* = 0.3
eGFR (ml/min/1.73 m^2^)	89 (56 – 109)	81 (54 – 114)	70 (47 – 102)	*p* = 0.2
<15 or RRT	12%	24%	15%	*p* = 0.1
15 – 49	19%	11%	19%	*p* = 0.4
50 – 90	32%	24%	39%	*p* = 0.1
>90	37%	40%	28%	*p* = 0.2
Vancomycin TDD (mg)	2000 (2000 – 2250)	2000 (1250 – 3000)	2000(1250 – 2500)	*p* = 0.5

Data presented as proportions or median (interquartile range). eGFR = estimated glomerular filtration rate; RRT = renal replacement therapy; TDD = total daily

**Table 3.** Proportion of vancomycin troughs in therapeutic (10 – 20 mcg/ml) range in historical controls, nomogram non-users, and nomogram-dosed patients at the extremes of weight and renal function.

	Controls	Non-users	Intervention	*p*-value
	(n = 75)	(n = 70)	(n = 108)	
Actual bodyweight (kg)				
<60	4/10 (40%)	5/14 (36%)	9/11 (82%)	*p*= 0.05
60 – 80	6/19 (32%)	7/14 (50%)	36/50 (72%)	*p*= 0.01
81 – 100	18/30 (60%)	10/20 (50%)	14/23 (61%)	*p*= 0.7
>100	10/16 (63%)	14/22 (64%)	16/24 (67%)	*p*= 0.9
eGFR (ml/min/1.73 m^2^)				
<15 or RRT	5/9 (56%)	9/17 (53%)	12/16 (75%)	*p*= 0.4
15 – 49	8/14 (57%)	6/8 (75%)	14/20 (70%)	*p*= 0.6
50 – 90	12/24 (50%)	9/17 (53%)	28/42 (67%)	*p*= 0.4
>90	13/28 (46%)	12/28 (43%)	21/30 (70%)	*p*= 0.08

eGFR = estimated glomerular filtration rate; RRT = renal replacement therapy.
